# Relationship of Inflammatory Markers and Metabolic Syndrome in Postmenopausal Women

**DOI:** 10.3390/metabo12010073

**Published:** 2022-01-13

**Authors:** Renata Vargas Sinatora, Eduardo Federighi Baisi Chagas, Fernando Otavio Pires Mattera, Luciano Junqueira Mellem, Ana Rita de Oliveira dos Santos, Larissa Pires Pereira, Ana Luíza de Carvalho Aranão, Elen Landgraf Guiguer, Adriano Cressoni Araújo, Jesselina F. dos Santos Haber, Leila Campos Guissoni, Sandra Maria Barbalho

**Affiliations:** 1 Postgraduate Program in Structural and Functional Interactions in Rehabilitation, University of Marilia (UNIMAR), Marília 17525-902, SP, Brazil; renatasinatoravargas@hotmail.com (R.V.S.); efbchagas@gmail.com (E.F.B.C.); fernando_mattera@hotmail.com (F.O.P.M.); junqmellem@yahoo.com.br (L.J.M.); larissamepp@gmail.com (L.P.P.); analuiza.aranao@gmail.com (A.L.d.C.A.); elguiguer@gmail.com (E.L.G.); adrianocressoniaraujo@yahoo.com.br (A.C.A.); guissoni.campos@gmail.com (L.C.G.); 2 Interdisciplinary Center on Diabetes (CENID), University of Marilia (UNIMAR), Marília 17525-902, SP, Brazil; haber.jesselina@gmail.com; 3 Postgraduate Program, Faculty of Medicine of Marília (FAMEMA), Marília 17519-030, SP, Brazil; 4 Department of Biochemistry and Pharmacology, School of Medicine, University of Marília (UNIMAR), Avenida Higino Muzzi Filho, 1001, Marília 17525-902, SP, Brazil; anarsantos@hotmail.com; 5 School of Food and Technology of Marilia (FATEC), Marília 17500-000, SP, Brazil

**Keywords:** menopause, visceral fat, inflammation, IL-6, IL10, TNF-α

## Abstract

The increased deposition of visceral fat in the postmenopause period increases the production of inflammatory cytokines and the release of tumor necrosis factor- α (TNF-α), interleukin-6 (IL-6), and decrease in IL-10. This study investigated the relationship between inflammatory biomarkers and metabolic syndrome (MS) in postmenopausal women considering different diagnostic criteria. We conducted a cross-sectional observational study based on STROBE. Data were collected regarding the diagnostic criteria for MS (International Diabetes Federation; NCEP (International Diabetes Federation (IDF), National Cholesterol Education Program Adult Treatment Panel III (NCEP ATP-III), and Harmonized criteria), body composition, comorbidities, time without menstruation, values of IL-6, IL-10, and TNF-α. ANOVA, Kruskal–Wallis, Levene tests, ROC, and odds ratio were performed to analyze the data. The results showed no significant difference between the methods and no interaction between the method and the presence of MS. However, for the values of WC, body fat percentage, TNF-α, and IL-10/TNF-α ratio, a significant effect of MS was observed. In subjects with MS, lower values of body fat percentage and TNF-α and higher values of the IL-10/TNF-α ratio were also observed. The higher IL-10/TNF-α ratio in the MS group is related to the greater anti-inflationary action of IL-10. The IL-10/TNF-α ratio showed significant accuracy to discriminate patients with MS according to the NCEP-ATP III criteria.

## 1. Introduction

Metabolic syndrome (MS) is a multifactorial metabolic condition currently considered one of the leading public health problems in women and men worldwide, with smiting rates of 30% in some populations (20–25% in western countries). There are some definitions and diagnostic consensuses for MS, such as NCEP ATP-III (National Cholesterol Education Program Adult Treatment Panel III), IDF (International Diabetes Federation), and Harmonized criteria. The harmonized criteria were created to bring harmony among several existing definitions of the MS [1,2,3,4,5,6]. These criteria comprise cardiometabolic risk factors such as dyslipidemia, hyperglycemia, obesity, and hypertension. These risk factors are related to a sedentary lifestyle, high sugar and fat, dietary habits, and stress, leading to a pro-inflammatory and pro-oxidative state [7,8,9,10].

The incidence rates for MS augment significantly with age, mainly in people aged over 50 (40–45%). Besides, modifications in the homeostasis regarding insulinotropic and anti-inflammatory cytokines due to an increase in the deposition of visceral fat can result in insulin resistance, which is one of the components of the definition of MS. Moreover, several studies have found that postmenopausal women are more vulnerable to present MS [11,12,13,14].

In postmenopause, weight gain and central fat accumulation are commonly observed mainly due to the imbalance in estradiol signaling leading to dyslipidemia, insulin resistance/type 2 diabetes mellitus (DM2), hypertension, and cardiovascular disease (CVD) [15,16,17,18,19,20,21,22]. The increased deposition of visceral fat increases the production of inflammatory cytokines, generating a low-grade inflammatory state and the release of tumor necrosis factor-α (TNF-α) and interleukin-6 (IL-6), as well as a decrease in IL-10, which is considered to be anti-inflammatory.

Adipocyte dysfunction, a common feature of MS, is associated with an increase in the M1 macrophage population in adipose tissue. This scenario can result in increased secretion of IL-6 and TNF-α that may contribute to the various disease processes associated with MS, precisely because of its systemic action. IL-10 is a predominantly anti-inflammatory cytokine that plays a role in modulating systemic inflammation. One of its functions is to help promote normal tissue remodeling following an inflammatory response. By antagonizing the pro-inflammatory actions of IL-6 and TNF-α, IL-10 seems to exert a protective effect against the increase in these cytokines [23,24,25]. 

The ovarian dysfunction in postmenopause may contribute to the increase of pro-inflammatory cytokines. Some, such as TNF-α, seem to aggravate hypertension associated with hyperglycemia, visceral obesity, and dyslipidemia to configure the MS scenario [24,25,26,27,28,29,30].

Given the above, the aim of this study was to investigate the relationship between inflammatory biomarkers and MS in postmenopausal women considering different diagnostic criteria. Furthermore, we also intended to analyze the performance of cutoff points for IL-10, IL-6, and TNF-α in the diagnosis of MS.

## 2. Results

Table 1 presents descriptive data on age, time without menstruation, body composition, blood pressure, glycemia, lipid profile, and inflammatory markers that characterize the sample. Regarding morbidity, the most prevalent was dyslipidemia.

Table 2 shows the prevalence of diagnostic criteria for MS, according to the harmonized, IDF, and NCEP-III criteria. The NCEP-III had the lowest prevalence (25.7%) when compared to the harmonized (38.6%) and IDF (37.1), but with no statistically significant difference according to the analysis of 95% confidence intervals. However, a significant difference was observed between the diagnostic criteria for abdominal obesity (waist circumference). The NCEP-III criterion showed a prevalence of abdominal obesity of 67.1%, significantly lower than the harmonized (94.2%) and IDF (94.2%) criteria by the 95% confidence intervals analysis. Abdominal obesity was the most prevalent factor in the diagnosis of MS, regardless of the criteria used.

Table 3 presents the analysis of the performance of cutoff points for inflammatory markers and their reasons for identifying the presence of MS by different diagnostic methods. The study of the area under the curve (AUC) showed that the inflammatory markers could not identify the subjects who have MS, except for the IL-10/TNF-α ratio for the NCEP ATP-III method, which showed a sensitivity of 72.2% and specificity of 63.4%. Although without statistical significance, IL-6 > 2.17 (pg/mL) for the harmonized and IDF methods, and values higher than 2.56 (pg/mL) for the NCEP ATP-III method were the inflammatory markers with the most heightened sensitivity for the diagnosis of MS. High sensitivity values were also observed for IL-6 in the three criteria (85.1, 84.6, and 72.2, respectively, for harmonized, IDF, and NCEP ATP-III). Moreover, although not significant, it is possible to observe that IL-10 shows a sensitivity of 72.2 and specificity of 61.5 for NCEP ATP-III criteria. High specificity values were observed for TNF-α (79.0, 79.5, and 80.7, respectively, for harmonized, IDF, and NCEP ATP-III) and the ratio IL-10/IL-6 in the NCEP ATP-III criteria (88.4).

Table 4 compares the values of inflammatory markers and their ratios between subjects with and without MS for the different diagnostic methods. There was no significant difference between the methods and no interaction between the method and the presence of MS. However, for the values of WC, body fat percentage, TNF-α, and IL-10/TNF-α ratio, a significant effect of the presence of MS were observed. In subjects with MS, lower values of body fat (%) and TNF-α and higher values of the IL-10/TNF-α ratio were also observed.

## 3. Discussion

Moreover, a record of dietary behavior was carried out in the sample. Daily caloric intake averaged 1407 ± 300 (kcal/day). Considering the resting metabolic rate of the mean sample of 1414 ± 144 (kcal/day), we can assume that the dietary pattern was in energy balance. Regarding the distribution of macronutrients in the diet, 62.1 ± 7.8% of carbohydrates, 22.1 ± 6.3% of protein, and 15.8 ± 3.8% of lipids were observed, which can be considered an adequate diet.

For the variables that showed a significant effect for two-way ANOVA, the effect size was estimated using Eta^2^ values and the study power. The main effect of the presence of MS was verified for the variables WC, % fat, TNF-α, and IL-10/ TNF-α (Table 4). The Eta^2^ values indicate a small effect size for WC (Eta^2^ = 0.026), % fat (Eta^2^ = 0.025), TNF-α (Eta^2^ = 0.037), and IL-10/TNF-α (Eta^2^ = 0.038). The observed power analysis indicates that the study has an adequate power to identify significant differences for WC (power = 0.647), % fat (power = 0.626), TNF-α (power = 0.799), and IL-10/TNF-α (power = 0.809).

As commented above, the NCEP-III had the lowest prevalence for MS when compared to the harmonized and IDF criteria. Following the American Heart Association/National Heart, Lung, Blood Institute update of the ATP III MS [31] and the IDF [4], the primary objective for diagnosing the presence of MS is to identify subjects with high long-term risk of developing CVD and DM2. Based on the diagnostic it is possible to perform adequate therapies to reduce these risk factors [32]. Due to increased life expectancy, identifying the presence of MS in postmenopausal women can ensure a better quality of life and lower risk of cardiovascular complications.

Assman et al. [33] investigated the prevalence of MS using the NCEP ATP-III and the IDF criteria in two American and 1 German population and found that, when the first criteria were used, the prevalence of MS was higher in both men and women in the United States than in German samples, but, when they used the IDF criteria the opposite occurred. In our sample, we did not observe differences in postmenopausal women using these criteria (25.7% when NCEP ATP-III was used and 37.1 when IDF was used) or using the harmonized criteria (38.6%). However, it is relevant to mention that the prevalence of MS in the studied population is high in any of the three criteria (mainly for the Harmonized). Although not significant, the NCEP criterion had a lower prevalence of MS, which is related to differences in the cutoff point for waist circumference.

Pokharel et al. [5] also compared the prevalence of MS using NCEP ATP-III, IDF, and harmonized definitions in Nepalese DM2 patients and found rates of 73.9%, 66.8%, and 80.3%. Moreover, according to these authors, the NCEP ATP-III definition could identify fewer patients with central obesity in a diabetic subject’s sample due to WC’s very high cutoff points. In our study, the higher prevalence for MS in postmenopause women was found in the harmonized definition (38.6%), and the lower was observed in the NCEP ATP-III (25.7%) (Table 2). 

Another study [33] also investigated the three above-mentioned criteria to investigate the presence of MS in diabetic patients and found 72.7%, 50.2%, and 53.9%, respectively, in harmonized criteria, NCEP ATP-III, and IDF. The authors concluded that elevated body mass index, dyslipidemia, and hypertension were the most relevant predictive risk factors of MS. Moreover, the maximum prevalence of MS was seen when the harmonized criteria were performed.

The use of the harmonized and IDF criteria appears to be more suitable for postmenopausal women due to lower waist circumference cutoff values. In the present study, when considering the NCEP criteria for waist circumference, 32.9% did not present abdominal obesity, but the fat percentages were high (>35%). Elevated body mass index, dyslipidemia, and hypertension, besides the hormonal modifications observed in postmenopause, are associated with increasing white adipose tissue. These metabolic alterations are related to increased release of inflammatory markers leading to a low-grade inflammatory state promoted by augmented serum levels of IL-6 and TNF-α and reduction in IL-10 [26,27,28,29,30,34]. A chronic low-grade inflammatory state is evident in postmenopause women, and controlling this condition can be relevant for the prevention of the most common diseases in women [35].

TNF-α and IL-6 are essential inflammatory biomarkers secreted by lymphocytes and macrophages. Besides estrogen decrease in postmenopausal women, their elevation is associated with chronic diseases such as dyslipidemia, high blood pressure, and CVD. Moreover, estrogen stimulates the synthesis of IL-6 and TNF-α [36,37,38]. Zannas et al. [39], advocating that cardiometabolic risks are augmented due to the psychosocial stress exposition and during the menopausal transition in women, investigated the role of IL-6 in predicting longitudinal cardiometabolic outcomes in women at perimenopause. The findings of these authors showed that higher levels of IL-6 can predict longitudinal elevation in these metabolic risks in perimenopausal women and also evidence a link between systemic inflammation with MS. In our study, we cannot say that IL-6, TNF-α, and IL-10 can identify subjects with MS, except for the IL-10/TNF ratio. Therefore, without significance, the IL-6 values are sensitive to identifying MS in the three different criteria (harmonized, NCEP ATP-III, and IDF). 

Santos–Marcos et al. [40] showed higher IL-6 and TNF-α levels in healthy postmenopausal women compared to the premenopausal state. Our results showed no significant difference between the methods and the presence of MS, but a significant effect was seen for the values of WC, body fat percentage, TNF-α, and the IL-10/TNF-α ratio. In subjects with MS, lower values of body fat percentage and TNF-α and higher values of the IL-10/TNF-α ratio were also observed. Lower TNF-α values in subjects with MS may be related to lower body fat. Although not significant, subjects with MS had higher IL-10 and IL-6 values and a lower IL-10/IL-6 ratio. We hypothesized that, although IL-10 is identified as an anti-inflammatory marker, subjects with MS had augmented values, which may be related to a higher inflammatory state associated with higher values of IL-6 in these MS subjects. On the other hand, lower TNF-α values in subjects with MS may be related to higher IL-10 values. It is noteworthy that although the group with MS had a significantly lower fat percentage than the group without MS, the mean values of fat percentage in the group with MS are high (>50% fat), which strongly contribute to high values of IL-6. The interaction between estrogen–progesterone and inflammation biomarkers can explain the higher inflammation process observed during hormonal fluctuation periods. Thus, reducing the IL-6 levels induced by progesterone can decrease the tissue inhibitor of metalloproteinases and augment the activity of proteolytic enzymes and high levels of TNF-α, leading to inflammation and clinical manifestations [41]. Some authors suggest that IL-10 exerts a protective effect against the increase in IL6 and TNF, as an antagonist to the pro-inflammatory actions of IL-6 and TNF. High levels of IL-10 are significantly correlated with the same levels of other cytokines such as IL-6 and TNF [24,25,42].

A relevant achievement of medicine is the increased lifespan of women. However, the comorbidities of postmenopause and increasing aging have impacted the health systems. The challenge of researchers, physicians, and the government is to improve the health span, quality of life and reduce the burden to health systems. For these reasons, the knowledge regarding the prevention of MS and CVD is of paramount importance. Our results can help researchers develop new approaches to investigate and treat MS in postmenopausal women, helping reduce a near-future consequence. 

This is the first study comparing the IDF, NCEP ATP-III, and harmonized definitions for MS in postmenopausal women to the best of our knowledge. Moreover, this is the first study showing the role of IL-6, TNF-α, and IL-10 in these different criteria for MS identification. Although only the IL-10/TNF-α ratio showed significant accuracy considering the sensitivity and specificity criteria, IL-6 showed good sensitivity to diagnose MS, and TNF-α showed good specificity to identify those who do not have MS. IL-6 is also associated with each of the components of MS. In a study of postmenopausal women, elevated IL-6 was also associated with abdominal obesity, low HDL, and high triglycerides [43]. Moreover, it is worthy to say that other adipokines released by adipose tissue may interfere with the pathogenesis and treatment of MS. Strategies to increase adiponectin release, for example, can reduce risk factors and, consequently, reduce negative outcomes in postmenopausal women [44].

Despite the advances in medicine, MS and cardiovascular diseases are still high. The combination of a diet rich in antioxidants and anti-inflammatory compounds has a critical role in reducing oxidative stress and pro-inflammatory processes that are closely associated with endothelial dysfunction and the development of atherosclerotic plaques. The Mediterranean diet, for example, presents at least six classes of phenolic components with powerful antioxidant properties. Phytochemicals with present antioxidant properties are vitamins A, C, and E, selenium, zinc, carotenoids, flavonoids, and several other phenolic compounds [45]. Besides that, the use of isoflavones can be effective in correction and improving the lipid profile and metabolism in postmenopausal women and could favorably prevent cardiovascular events [46].

These results open an important perspective for further studies in postmenopausal women that investigate the potential of different inflammatory markers in the pathophysiology of MS. We can infer that TNF-α and IL-6 could be useful not only as markers but could help in monitoring MS, as well as in early intervention for postmenopausal MS.

## 4. Material and Methods

### 4.1. Type of Study and Included Patients

This is a cross-sectional observational study conducted based on the STROBE recommendations [47] that were approved by the Research Ethics Committee (according to the protocol 364/2011) and the COMAP (Municipal Committee of Evaluation and Research) (process 476/11-SS). The data collection period was from December 2011 to March 2012. Data were collected regarding the diagnostic criteria for MS, body composition, comorbidities, time without menstruation, use of medications, and serum values of IL-6, IL-10, and TNF-α. Data were collected in three sections as follows: first day: home visit and invitation to participate in the research; second day: initial assessment and identification of inclusion criteria; third day: blood collection for biochemical analysis. Three different diagnostic criteria for MS were considered.

The minimum sample size was estimated at 52 sample elements for a type I error margin of 1%, study power of 80%, and large effect size of 0.80 [48] for comparing means between independent groups.

The sample was made up of women aged between 50 and 79 years old, who met the following inclusion criteria: (a) absence of menstruation for at least five years; (b) body fat percentage (% BF) ≥ 35% (diagnosis of obesity) [49]; (c) not in a hormone therapy period; (d) not presenting physical incapacitation.

Data were obtained from a Family Health Unit (FHU) in the city of Marília—SP. In Brazil, cities have a basic health care structure called FHU, which are small health units distributed in neighborhoods and allow the registration of all people residing in the area of operation of each FHU. In our study, an FHU was randomly selected and a random sample of women who could meet the inclusion criteria was selected. Considering that a rigorous and appropriate methodology was used, we believe that the sample studied is representative of the population of the city where the research was carried out.

### 4.2. Anthropometric Parameters

Body mass index (BMI) was calculated by dividing weight by squared height (kg/m^2^). Waist circumference (WC) was obtained to evaluate the presence of abdominal obesity. Lean body mass and body fat were calculated by bioelectrical impedance analysis (and with specific equations for postmenopausal women) [50]. Fat percentage values ≥ 35% will be considered as obesity [33].

### 4.3. Biochemical Parameters

Biochemical parameters included the evaluation of fasting glycemia (FG), triglycerides (TG), total cholesterol (TC), very low-density lipoproteins (VLDL-c), low-density lipoprotein (LDL-c), high-density lipoprotein (HDL-c), TNF-α, IL-6, and IL-10. The subjects were instructed to fast and not perform physical activity in the previous 12 h and not take alcohol for 72 h. The evaluation of the levels of cytokines was obtained with the Enzyme-Linked Immuno-Sorbant Assay (ELISA) Kit (BD Biosciences) with a detection limit of 2.0 pg/mL for IL-10 and TNF-α, and 2.2 pg/mL for IL-6. The ratios between IL-10 and TNF-α (IL-10/TNF-α) and IL-10 and IL-6 (IL-10/IL-6) were evaluated to investigate the rate of synthesis between anti and pro-inflammatory biomarkers [51].

The presence of MS was evaluated according to NCEP ATP-III [3]; IDF (International Diabetes Federation) and harmonized criteria. NCEP ATP-III encloses the presence of any three of the following circumstances: hyperglycemia (fasting plasma glucose ≥ 100 mg/dL); waist circumference ≥ 102 cm in men, and ≥ 88 cm in women; serum HDL-c ≤ 40 mg in men, and ≤ 50 mg/dL in women; triglycerides ≥ 150 mg/dL; and blood pressure ≥ 130/85 mmHg. The IDF criteria considers the presence of abdominal obesity and any two of the following: waist circumference > 90 cm in men and > 80 cm in women; blood pressure > 130/85 mmHg; glycemia > 100 mg/dL; triglycerides > 150 mg/dL; HDL-c < 40 mg/dL in men and < 50 mg/dL in women. The harmonized criteria indicate the presence of MS in a patient with any of the three risk factors: waist circumference > 90 cm in men and > 80 cm in women; blood pressure > 130/85 mmHg; glycemia > 100 mg/dL; triglycerides > 150 mg/dL; HDL-c < 40 mg/dL in men and < 50 mg/dL in women [1,2,3,52,53].

### 4.4. Morbidities

Data on chronic diseases were obtained through anamnesis in the initial assessment and confirmed in medical records.

### 4.5. Statistical Analyses

Quantitative variables are described as mean and standard deviation (SD) or 95% confidence interval (95%CI). Qualitative variables are described by the distribution of absolute (f) and relative (%) frequency. Normality was analyzed using the Kolmogorov–Smirnov test and homogeneity of variances using the Levene test. Associations between qualitative variables were analyzed using the chi-square test. The means in the three diagnostic criteria were compared using one-way ANOVA or the non-parametric Kruskal–Wallis test. Post-hoc analyses were performed using the Bonferroni test. The Eta2 values determined the effect size for ANOVA. Eta2 values were interpreted as: 0.10 small; 0.25 medium; and 0.40 large [54]. The receiver operating characteristic curve (ROC curve) was used to identify the sensitivity and specificity of the cutoff points for inflammatory markers in determining the diagnosis of MS. The areas under the curve and the 95% CI were performed, and the 95% CI did not include the value of 0.50. The quantitative variables (inflammatory markers) were categorized and dichotomized for association analysis using the chi-square test and the odds ratio (odds) estimation for MS from the established cutoff points. The established significance was 5% (*p*-value ≤ 0.05), and the analyses were performed using SPSS software, version 24.0 for Windows.

## 5. Conclusions

The fat percentage and the lower TNF-α values in the groups with MS highlight the relationship between these variables. Higher IL-10/TNF-α ratio values in the MS group suggest that higher IL-10 concentrations may be contributing to the reduction of TNF-α in the MS group. Furthermore, the IL-10/TNF-α ratio showed significant accuracy to discriminate patients with MS according to the NCEP criteria.

## Figures and Tables

**Table 1:** Descriptive statistics of the variables that characterize the sample (*n* = 70).

Parameters	Mean	SD
Age (year)	60.54	6.74
Time without menstruation (months)	160.94	100.18
BMI (kg/m^2^)	31.78	5.21
WC (cm)	95.69	12.29
Lean mass (kg)	35.22	5.51
Fat (%)	54.88	3.18
SBP	128.66	13.15
DBP	81.94	8.95
Gl (mg/dL)	95.97	18.39
TC (mg/dL)	209.84	32.96
TG (mg/dL)	147.27	64.32
HDL-c (mg/dL)	53.57	11.82
LDL-c (mg/dL)	129.04	30.31
VLDL-c (mg/dL)	29.46	12.86
Non-HDL-c (mg/dL)	156.27	33.34
IL-6 (pg/mL)	3.95	2.72
TNF-α (pg/mL)	9.03	4.65
IL-10 (pg/mL)	12.30	4.44
IL10-IL6 (pg/mL)	4.20	2.54
IL10-TNF-α (pg/mL)	2.22	2.19
Condition	*n*	%
Dyslipidemia	46	65.7
Hypertension	43	61.4
Diabetes Mellitus	12	17.1
Osteoporosis	9	12.9
Osteoarthritis	21	30.0

BMI: body mass index; DBP: diastolic blood pressure; Gl: glycemia; HDL-c: high-density lipoprotein; IL: interleukin; LDL-c: low-density lipoprotein; *n*: absolute frequency; SD: standard deviation; SBP: systolic blood pressure; TC: total cholesterol; TG: triglycerides; TNF-α: tumor necrosis factor-α; VLDL-c: very low-density lipoprotein; WC: waist circumference; %: relative frequency.

**Table 2:** Frequency distribution (%) and 95% confidence interval (95% CI) of the presence of metabolic syndrome (MS) according to harmonized, IDF, and NCEP ATP-III criteria.

	Harmonized			IDF			NCEP ATP-III		
	%	CI 95%		%	CI 95%		%	CI 95%	
		LL	UL		LL	UL		LL	UL
WC	94.2 ^a^	86.2	97.8	94.2 ^a^	86.2	97.8	67.1 ^b^	55.5	77.0
BP (mmHg)	22.9	14.6	34.0	22.9	14.6	34.0	22.9	14.6	34.0
GL (mg/dL)	25.7	16.9	37.0	25.7	16.9	37.0	18.6	11.2	29.2
TG (mg/dL)	37.1	26.8	48.9	37.1	26.8	48.9	37.1	26.8	48.9
HDL (mg/dL)	44.3	33.2	55.9	44.3	33.2	55.9	44.3	33.2	55.9
MS	38.6	28.0	50.3	37.1	26.8	48.9	25.7	16.9	37.0

BP: blood pressure; GL: glycemia; HDL-c: high-density lipoprotein; IDF: International Diabetes Federation; lower limit (LL); MS: metabolic syndrome; NCEP-ATP III: The National Cholesterol Education Program ATP III criteria; TG: triglycerides; upper limit (UL). Note: Different superscript letters indicate a significant difference in prevalence between methods based on 95% CI analysis.

**Table 3:** Analysis of the performance of cutoff points for IL-10, IL-6, TNF-α, IL-10/IL-6, and IL-10/TNF-α for the diagnosis of metabolic syndrome by different diagnostic criteria.

Criteria	Parameter	Cutoff Point for MS	AUC (IC95%)	*p*-Value	Sensitivity (IC95%)	Specificity (IC95%)
Harmonized	IL-10 (pg/mL)	>12.23	0.56 (0.44–0.68)	0.34	59.2 (38.8–77.6)	60.4 (44.4–75.0)
IDF		>12.23	0.56 (0.43–0.67)	0.40	57.6 (36.9–76.6)	59.0 (43.2–73.7)
NCEP ATP-III		>12.23	0.61 (0.48–0.72)	0.13	72.2 (46.5–90.3)	61.5 (47.0–74.7)
Harmonized	IL-6 (pg/mL)	>2.17	0.55 (0.42–0.67)	0.45	85.1 (66.3–95.8)	41.8 (27.0–57.9)
IDF		>2.17	0.53 (0.41–0.65)	0.62	84.6 (65.1–95.6)	40.9 (26.3–56.8)
NCEP ATP-III		>2.56	0.57 (0.45–0.69)	0.35	72.2 (46.5–90.3)	48.0 (34.0–62.4)
Harmonized	TNF-alfa (pg/mL)	≤6.39	0.60 (0.47–0.71)	0.15	44.4 (25.5–64.7)	79.0 (64.0–90.0)
IDF		≤6.39	0.60 (0.47–0.71)	0.16	46.1 (26.6–66.6)	79.5 (64.7–90.2)
NCEP ATP-III		≤5.24	0.63 (0.50–0.74)	0.09	50.0 (26.0–74.0)	80.7 (67.5–90.4)
Harmonized	IL-10/IL-6 (pg/mL)	>7.68	0.51 (0.39–0.63)	0.83	3.7 (0.09–19.0)	86.0 (72.1–94.7)
IDF		>3.43	0.53 (0.40–0.65)	0.66	61.5 (40.6–79.8)	54.5 (38.8–69.6)
NCEP ATP-III		>6.51	0.51 (0.38–0.63)	0.91	27.7 (9.7–53.5)	88.4 (76.6–95.6)
Harmonized	IL-10/TNF-α (pg/mL)	>1.41	0.61 (0.49–0.73)	0.09	59.2 (38.8–77.6)	62.7 (46.7–77.0)
IDF		>4.26	0.61 (0.49–0.73)	0.10	30.7 (14.3–51.8)	88.6 (75.4–96.2)
NCEP ATP-III		>1.41	0.67 (0.54–0.78)	0.01 *	72.2 (46.5–90.3)	63.4 (49.0–76.4)

IDF: International Diabetes Federation; lower limit (LL); IL: interleukin; MS: metabolic syndrome; NCEP-ATP III: The National Cholesterol Education Program ATP III criteria; TNF-α: tumor necrosis factor-α; upper limit (UL). Note: Area under the curve (AUC); 95% confidence interval (95%CI); * indicates a significant cutoff effect for the diagnosis of MS; the cutoff point was established by the confidence interval of the Youden index.

**Table 4:** Comparison of mean 95% confidence intervals (95%CI) of waist circumference (cm), body fat (%), and inflammatory markers between subjects with and without metabolic syndrome (MS) for the three diagnostic methods.

Parameter	MS	Criteria									Two-Way ANOVA		
		Harmonized			IDF			NCEP ATP-III					
		Mean	CI95%		Mean	CI95%		Mean	CI95%		MS	Criteria	Interaction
			LL	UL		LL	UL		LL	UL	*p*-Value ^a^	*p*-Value ^b^	*p*-Value ^c^
WC (cm)	No	94.6	90.7	98.5	94.7	90.9	98.5	93.8	90.5	97.1	0.020 *	0.768	0.515
	Yes	97.4	92.7	102.0	97.4	92.5	102.2	101.1	95.0	107.1			
Fat (%)	No	55.4	54.4	56.3	55.4	54.5	56.3	54.9	54.1	55.8	0.023 *	0.983	0.569
	Yes	54.0	52.7	55.3	53.9	52.6	55.2	54.6	52.8	56.3			
IL-6 (pg/mL)	No	3.90	3.03	4.77	3.95	3.10	4.81	3.78	3.04	4.51	0.520	0.939	0.779
	Yes	4.02	3.00	5.05	3.94	2.89	5.00	4.45	2.96	5.93			
TNF-α (pg/mL)	No	9.70	8.31	11.09	9.69	8.33	11.04	9.60	8.32	10.87	0.005 *	0.909	0.953
	Yes	7.96	6.08	9.85	7.91	5.96	9.87	7.38	5.10	9.66			
IL-10 (pg/mL)	No	12.00	10.56	13.44	12.02	10.62	13.43	11.92	10.67	13.17	0.127	0.934	0.886
	Yes	12.79	11.18	14.40	12.77	11.10	14.45	13.40	11.30	15.50			
IL10/IL6 (pg/mL)	No	4.29	3.42	5.17	4.25	3.39	5.11	4.22	3.49	4.94	0.739	0.999	0.979
	Yes	4.06	3.28	4.85	4.14	3.33	4.94	4.17	2.98	5.37			
IL10/TNF-α (pg/mL)	No	1.88	1.30	2.46	1.87	1.30	2.44	2.00	1.40	2.60	0.005 *	0.959	0.994
	Yes	2.77	1.77	3.77	2.82	1.79	3.86	2.87	1.76	3.98			

IDF: International Diabetes Federation; IL: interleukin; MS: metabolic syndrome; NCEP-ATP III: The National Cholesterol Education Program ATP III criteria; TNF-α: tumor necrosis factor-α. Note: ^a^ *p*-value for the two-way ANOVA test for the main effect of the presence of MS regardless of the diagnostic criteria (method); ^b^ *p*-value for the two-way ANOVA test for the main effect of the difference between the diagnostic criteria (method) regardless of the presence or absence of MS; ^c^ *p*-value for the two-way ANOVA test for the effect of interaction between diagnostic criteria (method) and the presence or absence of MS; * indicates a significant difference between subjects with and without MS by the two-way ANOVA test for *p*-value ≤ 0.05.

## Data Availability

Data is contained within the article.
